# A Treatise on the Nature of Seminal Diseases, Impotency, and Other Kindred Affections; A Complete Practical Work on the Nature and Treatment of Venereal Diseases

**Published:** 1848-11

**Authors:** 


### BIBLIOGRAPHICAL NOTICES.

*A Treatise on the Nature of Seminal Diseases, Impotency, and other kindred affections; with practical directions for the management and removal of the cause producing them; together with hints to young men.* Illustrated by numerous engravings. By Homer Bostwick, Surgeon. Second edition. 12mo. pp. 251. New York, 1848.

*A complete practical work on the Nature and Treatment of Venereal Diseases, and other affections of the genito-urinary organs of the Male and Female.* Illustrated by a great number of beautifully coloured plates, and many finely executed delineations on wood. By Homer Bostwick, M. D., author of a Treatise on the Nature and Treatment of Seminal Diseases, &c. &c. &c. 4to. pp. 348.

It has been a serious question with us, whether we ought to notice these productions, proceeding, as they do, from an author whose advertisements for practice are prominently displayed in the columns of the New York newspapers. They have been sent to us, however, for comment, and we, consequently, esteem it a duty, for more reasons than one, not to pass them by unheeded.

The first of the volumes is manifestly written for the unprofessional, as well as for the professional reader. "The sick, however," says the author, "must not expect to find in this volume a key by means of which they can successfully treat themselves. It is impossible to furnish the unprofessional reader a work that will allow him to dispense with the services of the experienced surgeon."

We have looked over the pages and find great inaccuracy of statement on points on which every tyro is expected to be informed. Thus, speaking of the semen, the author says:

"In castrated animals, and in eunuchs, the vesiculæ seminales are small and contracted; and a little *lymphatic liquor*, but no semen is found in them. The semen is detained for some time in the vesiculæ seminales, and rendered thicker from the continual absorption of its very thin part, by the *oscula* of the lymphatic vessels." p. 22.

What is meant by a "little lymphatic liquor," and by the "oscula of lymphatic vessels," to which no one now concedes open mouths?

In chaste men—we are informed—the greatest part of the semen "is again gradually absorbed from the vesiculæ seminales through the lymphatic vessels, and *conciliates* strength to the body."—"The smell of the semen is specific, heavy, *affecting the nostrils*, yet not disagreeable."—"The smell of the semen of quadrupeds, when *at* heat, is so penetrating as to render their flesh fœtid and useless, unless (qu. the flesh) castrated."—"The taste of semen is *fatuous*, and somewhat acrid. In the testes its consistence is thin and *diluted*. Semen, when exposed to the air, at length "deposits phosphate of lime and a *corneous* crust."—"By æthereal oil it [semen] is dried into a pellucid pellicle, *like the cortex of the brain.*"

The author is a believer in the exploded doctrine of impregnation by the aura seminis, and other equally strange notions:—

"The *odorous principle*, or aura spermatica, only appears to penetrate through the cavity of the uterus and Fallopian tubes to the female ovarium, and there to impregnate the albuminous latex of the mature ovulum by its vital power. The other principles of the semen appear to be only a vehicle of the seminal aura. In chaste men, the semen returning through the lymphatic vessels into the mass of the blood, gives strength to the body and mind; hence the bull is so fierce and brave, the castrated ox so gentle and weak; hence every animal languishes *post coitum*."

The spermatic "animalculæ" (*animalcula*) were discovered—we are told—by a student at Leyden named "*Horn*" (?) In erection the male organ "*swells* and distends in all directions. It becomes *swollen*, stiff and hard."

The following sentence must be ranked amongst the palpably obscure:

"The ovaria are two small bodies formed of two membranous envelopes, and a fluid which runs into a mass, and becomes hardened like albumen. They are situated on each side of the womb, in the cavity formed by the bony structure called the *pelvis*. These vessels [qu. ovaries] appear to contain the rudiments of the germ, and to bear to women the same relation that the eggs do to birds, fishes, and reptiles."

Women, who are barren from want of development of the ovaries, are described as having "a harsh voice, hair on the chin and lips, and want of feminine delicacy of form. The clitoris, in such cases, is unnaturally large, and *inclinations manifest themselves, which has given rise* to the idea of hermaphrodism in many cases, where such a supposition is not correct."

"The most favourite theory" [of fecundation] "has been, that the uterus or womb absorbs the semen, and directs it to the ovaria through the Fallopian tubes; and that one of *their vesicles* being ruptured, the *fluid which escapes*, or the vesicle itself, is *carried back* to the womb, where the germ becomes developed."

"On the whole, then,"—the author concludes—"we must rest content, that this is one of the mysteries of our nature, which we can never fathom. Like the *rationale* of vegetating and other marvellous productions, *the fecundation of the human uterus* lies, and must be, far beyond our ken."

The author then proceeds to arrange and classify, under their appropriate heads, the different species of seminal disease; for which, he informs us, "the widely extended experience" he has "enjoyed in the management of the 'Medical and Surgical Institute,' has afforded ample materials." But we have presented extracts enough to render him full justice, as regards the style and accuracy of his materials; and we can afford space only to say, that in his large volume, he has been, on many points, more correct.

"The chapters describing the male and female organs of generation, have been selected mainly from Bell." He has evidently gone to a considerable outlay in regard to the numerous illustrations, some of which, he says, he has "borrowed from the great work of Ricord, others from Acton and Judd," and some he has had copied directly from life. "I have intended"—he remarks—"to make this the most elegant and accurate medical publication that has ever been issued from the American press, and I do not hesitate to say, that the plates will bear a favourable comparison with the finest artistical productions of London or Paris." We would not institute comparisons which, as Dogberry remarks, are "odorous," but every one must admit that most of the lithographic illustrations are creditable to the artist. Some of the wood-cuts are, however, contemptible.

We have been at a loss to determine whether this work also was not intended rather for the public. Perhaps the following advertisement, copied in fac simile from the New York Courier and Enquirer of the 21st ult., may tend to throw light on the subject.

DR. HOMER BOSTWICK,

LECTURER ON DISEASES OF THE GENITO-URINARY ORGANS.

*Author of a Complete Practical Work on the Nature and Treatment of Venereal Diseases.*

Quarto, 77 splendid plates.

Extracted from the Boston Medical and Surgical Journal :—"It is illustrated by extraordinary specimens of colored lithography, equalling copper engravings in the delicacy of their finish. And it is a striking evidence of the indomitable perseverance of a man who seems to possess an unconquerable energy that will enable him to leave his foot-prints in society, so that he will be spoken of when the memorials of him are only to be found in the libraries of those who know not that he was both censured and envied while living. Without attempting a minute comparison of this Venereal Guide, with European publications on the same family of diseases, it may be said FEARLESSLY, that this is DECIDEDLY, and WITHOUT QUALIFICATION, equal to any of them. The plates in Acton's Treatise are not equal to these. By contrasting the two volumes, both the matter and the manner of giving instruction, in our humble estimation, should be accorded to the New York press. Dr. Bostwick has done the profession a good service, and nothing can prevent this able work from being spread over the land, as the fact can neither be denied nor concealed, that he has produced a thorough, well-digested, systematic treatise, which far surpasses any thing of the kind, on this branch of practice, heretofore attempted in this country."

Author of a treatise on the nature and treatment of Spermatorrhœa or Seminal Diseases, resulting from self abuse.

Impotency, and other Kindred Affections, 251 pages, 14 plates.

Author of a work entitled Hints to Young Physicians.

Author of the Family Physician,

And Attending Physician and Surgeon to the New York Medical Institute, 75 Chambers street; removed to 504 Broadway, eight doors above Broome street,

Where he may be consulted any time between the hours of 8 o'clock in the morning and 9 in the evening, and where any of the above works may be had. Also for sale at 155, 161, 222, 252, 575, 633 Broadway.

The ususl monthly reports of this institution, enumerating cases cured, will not be published for the present, HOMER BOSTWICK, M. D., 504 Broadway.

